# Shale Gas, Wind and Water: Assessing the Potential Cumulative Impacts of Energy Development on Ecosystem Services within the Marcellus Play

**DOI:** 10.1371/journal.pone.0089210

**Published:** 2014-02-19

**Authors:** Jeffrey S. Evans, Joseph M. Kiesecker

**Affiliations:** 1 The Nature Conservancy, Fort Collins, Colorado, United States of America; 2 Department of Zoology and Physiology, University of Wyoming, Laramie, Wyoming, United States of America; 3 The Nature Conservancy, Fort Collins, Colorado, United States of America; Plymouth University, United Kingdom

## Abstract

Global demand for energy has increased by more than 50 percent in the last half-century, and a similar increase is projected by 2030. This demand will increasingly be met with alternative and unconventional energy sources. Development of these resources causes disturbances that strongly impact terrestrial and freshwater ecosystems. The Marcellus Shale gas play covers more than 160,934 km^2^ in an area that provides drinking water for over 22 million people in several of the largest metropolitan areas in the United States (e.g. New York City, Washington DC, Philadelphia & Pittsburgh). Here we created probability surfaces representing development potential of wind and shale gas for portions of six states in the Central Appalachians. We used these predictions and published projections to model future energy build-out scenarios to quantify future potential impacts on surface drinking water. Our analysis predicts up to 106,004 new wells and 10,798 new wind turbines resulting up to 535,023 ha of impervious surface (3% of the study area) and upwards of 447,134 ha of impacted forest (2% of the study area). In light of this new energy future, mitigating the impacts of energy development will be one of the major challenges in the coming decades.

## Introduction

Global demand for energy has increased by more than 50 percent in the last half-century, and a similar increase is projected by 2030 [1]. Energy production to meet growing demand has resulted in impacts to human health and well-being as well as increased habitat fragmentation and stress on biological diversity worldwide [2]–[4]. In the United States, directives for renewable energy, energy security and technological advancements such as horizontal drilling in conjunction with hydraulic fracturing have spurred a rapid increase in alternative and unconventional energy production over the last decade [5]–[8]. Energy development is poised to continue its upward trajectory, with over 200,000 km^2^ of new land projected to be developed in the U.S. alone by 2035 [1], [4]. Development of “unconventional” gas dispersed in shale will be key, as the potential resource may be large [9]. In addition to shale gas, the U.S. Department of Energy's goals are to produce 20% of its electricity from terrestrial wind energy development (241 gigawatts of on-shore wind) by 2030 [8]. In light of this new energy future, understanding and mitigating the impacts of energy development will be one of the major challenges in the coming decades [10].

The Marcellus shale gas play covers approximately 160,934 km2 across eight states and contains both some of the largest technically recoverable shale gas resources is the U.S. and headwater watersheds that provide drinking water for over 22 million people in several of the largest U.S. metropolitan areas (e.g. New York City, Washington DC, Philadelphia & Pittsburgh) [11]. Land use change from shale gas and wind development is known to increase land clearing, impervious surface and increase deforestation[12]–[14]. Aquatic ecosystems are particularly vulnerable to land use change such as deforestation and change in impervious surface [11], [15], [16]. Deforestation and increases in impervious surface influence sediment, hydrologic, and nutrient regimes, which in turn influence aquatic biota and ecological processes in fresh waters [17]. Because sediment represents one of the most significant controlling variables on stream morphology and hydrology, deforestation and increases in impervious surface will be an important driver in stream health, integrity of headwater watersheds, and quality of drinking water. It is therefore essential to understand patterns of future deforestation and changes in impervious surface resulting from energy development.

Scenarios analysis has become a widespread approach in pursuit of sustainable development. However, it is used infrequently, at least in any formal way, in environmental impact assessment (EIA)[18]. This is surprising because EIA is a process designed specifically for exploring options for more-sustainable (i.e., less environmentally damaging) futures. Similarly predictive modeling techniques have been applied in recent years to project land cover changes and residential development [19]–[21] and to predict potential species habitat [22], [23], but comparable techniques are rarely used to model anticipated energy development and proactively quantify environmental impacts. Although other studies have estimated future development scenarios for portion of the Marcellus Shale [12], [24]–[26], this is the first to examine a comprehensive build out scenario for potential impacts associated with both shale gas and wind development across the entirety of the Marcellus Shale gas play.

Here we employ build-out scenarios for future energy development to quantify potential impacts on surface drinking water resources. First, we created prediction surfaces of wind and shale gas development potential for portions of six states in the Central Appalachians. Second, we modeled future build-out scenarios using these predictions and published projections from federal land management agencies [27]. Finally, we evaluated effects of build-out scenarios on ecosystem services by measuring impacts to watershed quality and identifying areas important for drinking water that are vulnerable to energy development. Studies have already documented potential point source impacts to surface water resources associated with shale gas fracking [13]. Here we focus on nonpoint source pollution across the entire landscape likely to change as a result of energy development [13], [14], [28]. Given expected development intensity, proactively addressing potential impacts can help develop strategies to mitigate issues related to biodiversity and surface water supply.

## Methods

### Study Area

**Figure 1:**
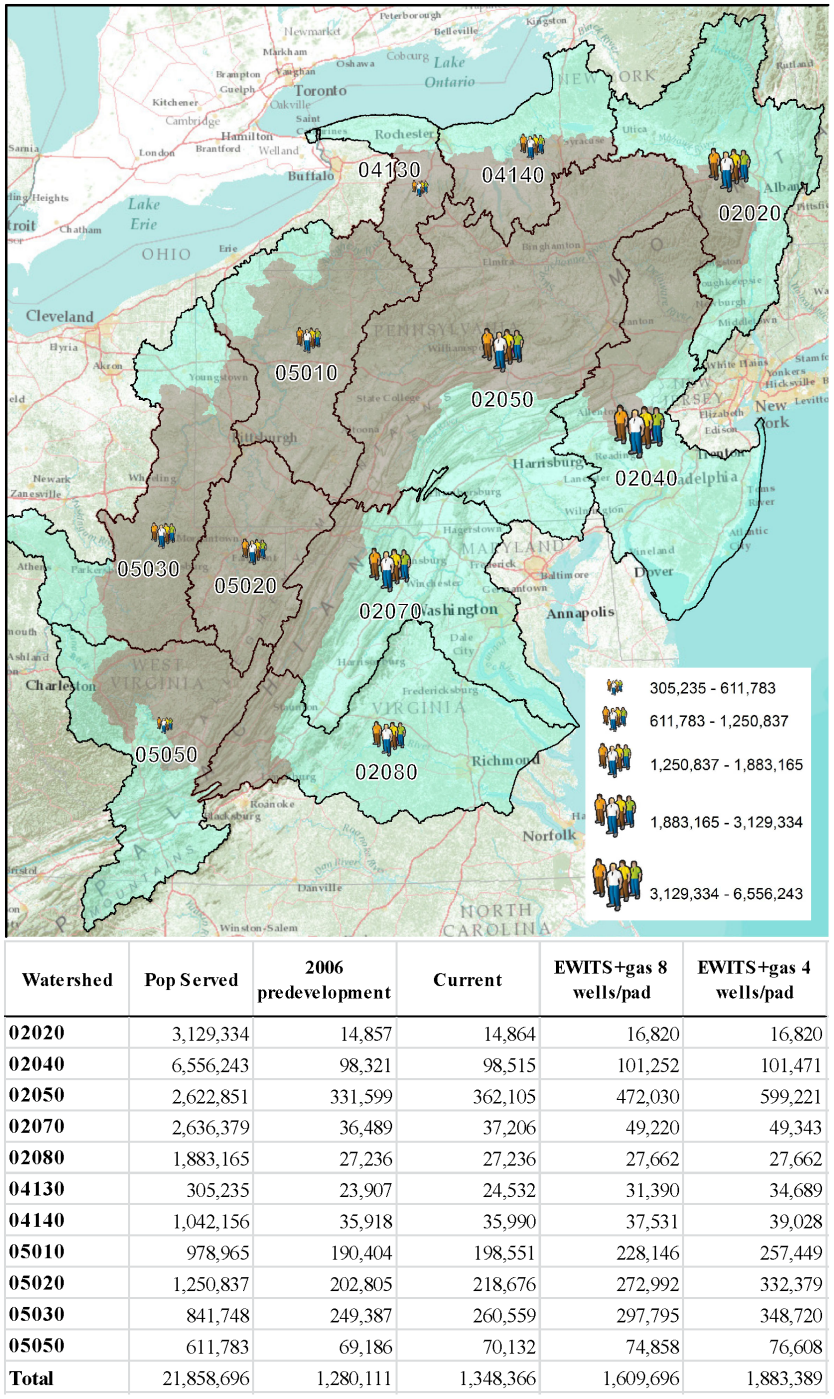
Study area with basin-level watersheds and population served, labels correspond to the watershed number. The interior polygon shaded in brown represented the subwatershed-level watersheds that intersect the high pressurization area that defines our study area. The inset table represents population served for each watershed, impervious surface for 2006 baseline, current and two scenarios. The hectares are specific to the given impact and are not cumulative. For total impacted area under a scenario, add baseline, current and scenario.

We focused our analysis on the Interior Marcellus Shale Assessment Unit (IMSAU) and defined the project boundary using the entirety of the “subwatershed” (12-digit) watersheds (*n* = 2003) that intersected the IMSAU (Figure 1). These subwatersheds, on average, are 8498 ha in size. The area comprises a complex landscape mosaic of 17,134,045 ha, with 12,007,150 ha (70%) of the area forested. There are currently 4151 well pads in the IMSAU with up to six wells drilled per pad for a total of 10,419 current and permitted wells. We compiled spatial databases of wind and gas development along with covariates identified, thorough a multidisciplinary scoping process, as influential to development (table 1).

**Table 1:** Variables used in the wind and gas models.

Variable	Description	Model
ASP	Linear transformation of slope direction	candidate wind
Bougure	Bougure gravity anomalies	included gas
CTI	Wetness index	candidate wind
Depth	Depth of shale	included gas
DPIPE	Distance in meters to nearest gas pipeline	candidate gas
DROADS	Distance in meters to nearest road	included wind, candidate gas
DTRANS	Distance in meters to nearest power transmission	included wind
ELEV	Elevation in meters	included wind
HLI	Heat Load Index	candidate wind
HSP	Slope position	candidate wind
Isograv	Isostatic gravity anomalies	included gas
Magnetic	Aeromagnetic gravity anomalies	included gas
PAG	Percent agriculture within 1 km radius	candidate wind
PDEV	Percent development within 1 km radius	candidate wind
PFOR	Percent forest within 1 km radius	candidate wind
SCOSA	Slope*COS(Aspect)	candidate wind
SLP	Slope intensity in degrees	included wind, candidate gas
SRR15	Surface Relief Ratio at 15×15 window	candidate wind, candidate gas
SRR27	Surface Relief Ratio at 27×27 window	included wind, considered gas
SRR3	Surface Relief Ratio at 3×3 window	candidate wind, candidate gas
TEX15	Variance of elevation at 15×15 window	candidate wind
TEX27	Variance of elevation at 27×27 window	candidate wind
TEX3	Variance of elevation at 3×3 window	included wind, candidate gas
Thickness	Shale thickness	included gas
Tmaturity	Geologic thermal maturity	included gas
WPC	Wind production classes	included wind

### Data sources

We compiled gas and wind spatial databases from private, state and federal sources. The magnetic anomaly rasters (bouguerbougure, isograv and magnetic) were obtained from the USGS [29] as was the thermal maturity point data [30]. We obtained point-locations of well monitoring data from U.S. Department of Energy, National Energy Technology Laboratory (Kathy Bruner, personal communication), which were compiled from state-level databases. The thermal maturity and well monitoring data were used to build ordinary Kriging models [31], using the geoR library [32] to generate 1-km rasters of thermal maturity, shale depth and shale thickness.

Turbine locations used in the wind model were obtained through the U.S. Federal Aviation Administration obstruction evaluation database. All topographic variables were derived from the 30 m Digital Elevation Model available through the USGS National Elevation Dataset. All topographic variables were derived in ArcGIS 10.0 using the Geomorphometrics toolbox [33]. We utilized the U.S. Census Bureau TIGER road data and land cover, including forest and impervious surface, was derived from the 2006 USGS National Land Cover Dataset [28]. Wind production class data was acquired from the DOE-NETL [8] and rasterized to 30 m. Spatial data representing energy infrastructure was obtained from Ventyx [50]. Protected areas, where development is jurisdictionally precluded, were determined using PADUS v 1.3 [34] with gap code A-2 (“statutory or legally enforceable protection”). Watersheds and stream data were acquired from the USGS National Hydrography Dataset (NHD) (http://nhd.usgs.gov/index.html). Finally, data associated with water access and production related ecosystem services were obtained through the U.S. Forest Service, Forests to Faucets analysis [35] and associated with the NHD.

### Statistical model

We implement a robust nonparametric model that provides stable spatial estimates of resource development probabilities. This is accomplished by independent model iteration with differing conditional random observations that act as the “absence” class in the model. The underlying model is an ensemble approach that allows us to combine model iterations into a final model after convergence is satisfied. Conditional random samples are generated by generating a Kernel Density Estimate (KDE) [36], using an isotropic Gaussian kernel intensity function in the R spatstat library [37] based on known resource development locations [38]. The inverse of this KDE is specified as the sample probability distribution for the NULL (i.e. absence observations) and conditional random samples are generated by using the probabilities as sample weights. The optimal KDE sigma parameter is selected using a cross validation.

The “positive” class is fixed and is represented by known observations. In a given iteration of the model, the number of random observations generated is the same size as the number known observations. A model is then built using Random Forests [39], [40], using the R library randomForest [41], and the probabilities are predicted back to the training data. The estimated probabilities are then set aside, a new set of random points created and the process is repeated. At each iteration, where the modeling process is repeated, the new model is combined with the previous ones and the new probability estimated based on the prior ensemble. This is repeated until the resulting probability distribution is unchanged, when compared to prior combined ensembles, using a Kolmogorov-Smirnov [42] distributional equality test (significant at p = 0.001). Once the model converges, it is predicted to the final set of raster surface variables identified in the model yielding a raster surface of the estimated probabilities. Since Random Forests is a weak learner ensemble method, as long as the parameter space remains fixed, multiple model ensembles (independent models) can be combined [43]. This method ensures that statistical and spatial variability is captured across the random samples used to represent the negative case. The parameter space is fixed across iterations and our model selection follows methods presented in Murphy et al. 2010 [44]. R Code is provided in appendix A.

### Development potential

The wind model was specified with a set of 17 candidate variables that are known to influence wind suitability and development (see Table 1) [8], [45]–[47]. Generally, these variables were related to topographic, anthropogenic, transmission and wind production influences. The model selection procedure retained 7 of the 19 variables (Table 1) used in the final model. To remove areas where wind development is precluded, pixel-level probabilities associated with proximity to airports; [12], protected areas based on the PADUS [34] gap status code 1 (“An area having permanent protection from conversion of natural land cover”), streams and roads were recoded to zero. The gas model was specified with 13 candidate variables (Table 1) that are known to influence oil and gas potential [12], [48]. Generally, these variables were related to geologic, topographic and current infrastructure characteristics. The final model retained six variables (Table 1) all associated with geological characteristics.

### Development scenarios

To calculate number of wells needed to exploit the IMSAU, we used a projection of gas development from U.S. Energy Information Administration [27], which predicts 141 trillion cu ft of gas with an estimated ultimate recovery of 1.15 billion cu ft/well. We designated well pads as our experimental unit so we could account for expected development reflecting the expansion of current leases (i.e., additional wells drilled on an already established well pad). We created scenarios around two potential intensities of well pad development: 4 and 8 wells per pad, for a respective total of 26,501 and 11,175 new well pads, which accounts for current well pad development. These scenarios have the current well pads subtracted (i.e., [(4 wells/pad = 26,501 * 4) + (4151 current well pads * 4)]  = 122,608 total wells) and are derived by solving for the maximum number of possible wells needed to exploit the resource, assuming that additional wells will first be drilled on established pads to reach the desired maximum density (4 or 8 wells per well pad). Local legal regulatory guidelines and lease availability were not addressed in our scenarios.

For the wind development scenario, we use projections from the Eastern Wind Integration and Transmission Study (EWITS) [49] and selected scenario 3 that emphasizes development of wind resources close to load centers in the northeastern US. This scenario represents an estimate on the high end of potential wind development for this region. We scaled the projection by the percentage of our study area intersecting the EWITS, using estimates of viable wind derived from wind production classes [8]. Taking currently installed capacity into account and, following the EWITS analysis, we estimate that our study area would need an additional 10,798 turbines to meet the EWITS projections. We assumed turbines would have a nameplate capacity of 2.5 MW and adjusted output (52%) which was consistent with the EWITS estimates and measures from current wind farm production compiled by Ventyx [50].

**Figure 2:**
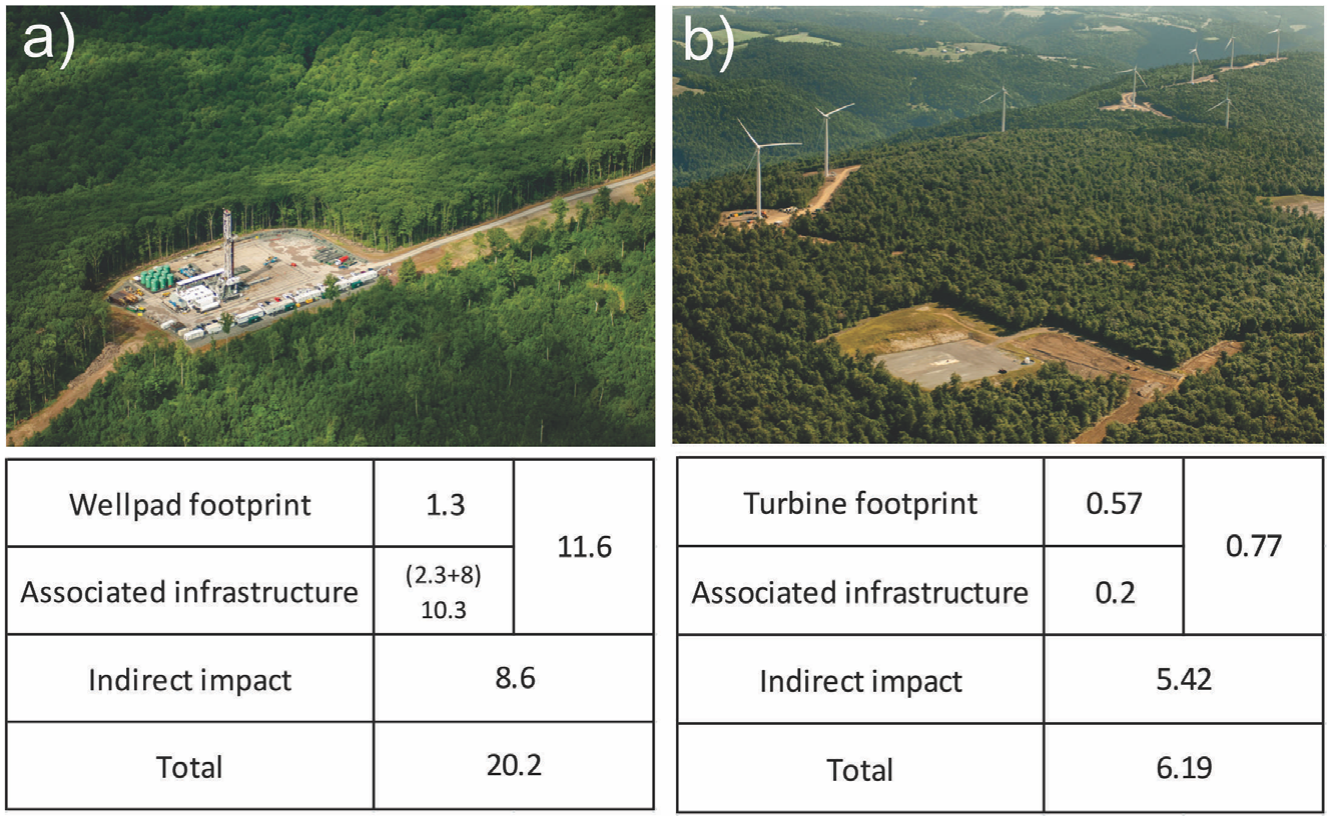
Photographs of shale gas footprint and wind farm footprint. Inset table represents associated impacts in hectares used in the analysis. Estimates of potential surface disturbance associated with gas wells and wind turbines were based on measurements taken from aerial photographs from Johnson (2010) and Johnson et al. (2011). We also incorporated impacts associated with the pipelines needed to collect gas from well sites and transport it to storage areas. Measurements indicate that on average there are 2.66 km of pipeline with an average right of way of 30.48 m, where each mile of a 30.48 m right-of-way directly disturbs ∼4.86 ha/well pad. The 8.019 ha of impact associated with offsite pipelines is included in the estimate of associated infrastructure for each well pad. Because we were unable to spatially configure the location of pipelines, we summed the surface disturbance associated with each simulated well in the watershed to estimate the amount of additional surface disturbance that pipelines would create at the watershed-level. Photographs by Mark Godfrey.

To create spatial data for a given wind or gas scenario, we generated random samples by iterating through the range of estimated sorted probabilities in the surface estimates, starting with the highest probability value(s), until the desired number of well pads or wind turbines is generated. We then take each simulated point location and buffer it to represent an expected footprint for each impact (Figure 2). Estimates of potential surface disturbance associated with gas wells and wind turbines were based on measurements of actual wind and shale gas development in Pennsylvania digitized from aerial photographs [12], [26]. These simulated footprints were then use to modify impervious surface and forest rasters to illustrate potential impacts.

### Surface impacts

Shale gas and wind development is known to increase land clearing, impervious surface and increase deforestation [12]–[14]. We evaluated effects of shale gas and wind energy development on ecosystem services by 1) measuring impacts to watershed quality based on imperviousness classifications [2], [51] and identifying vulnerable areas based on an importance index for drinking water production [35]. Current research suggests that aquatic systems become very seriously impacted when watershed impervious cover exceeds 10% [52] and show significant declines in many stream taxa at even lower levels of imperviousness [53]. For example, significant declines in species have been documented between 0.5 and 2% imperviousness, with 40–45% declines in regional stream biodiversity (invertebrates, fish, amphibians) at imperviousness greater than 2–3% [51], [53], [54]. Costs of water treatment have also been shown to increase in line with increases in impervious surfaces [51]. To examine the impact of impervious surface in our study area, we summarized the amount of current impervious cover for each subwatershed in the study area using the National Land Cover Impervious Surface Dataset [28], [55]. We grouped each subwatershed into one of four impact categories guided by the thresholds found in Schueler et al. [51]: Sensitive, 0<10% impervious; Impacted, 10–25% impervious; Nonsupporting, > = 25–60% impervious; Urban drainage, > = 60% impervious. We then used results from each development scenario to reclassify watersheds, and we summarized the percent change by impact class.

To quantify an area's value for surface drinking water, we utilized the Safe Drinking Water Information System, SDWIS [56] and mean annual drinking water supply [57] generated by the U.S. Forest Service's Forest to Faucets analysis [35]. The Forest to Faucets analysis produces an index of relative importance for water production, at the basin-level and subwatershed-level, which is based on the potential amount of water produced and the number of people who use that water. Since it is a measure of both production and population served, we use the metric as a measure of the ecosystem service. We highlighted forested areas important for drinking water production that might be susceptible to energy development impacts by identifying areas with both relative high water importance indexes (RIMP) scores and high percent forest cover (both at > = 0.75).

## Results

### Model validation

All Kriging models (shale depth, shale thickness and thermal maturity), used to generate covariates, exhibited a Kriging variance smaller than 1 standard deviation of the predicted distribution, illustrating very good performance. Both the gas and wind models were very well supported. The AUC/ROC for the wind model was 0.98 with a sensitivity weighted Kappa [58] of 0.97. The ROC for the shale gas model is 0.93, indicating a very strong model. The sensitivity weighted Kappa is 0.93, demonstrating that a random chance and bias corrected percent correctly classified also supports the model. In the shale gas models, when probabilities were assigned back to the training and validation observations, we see 90% of the observations above p = 0.65 for the training data and 84% in the validation data.

### Surface impacts

**Figure 3:**
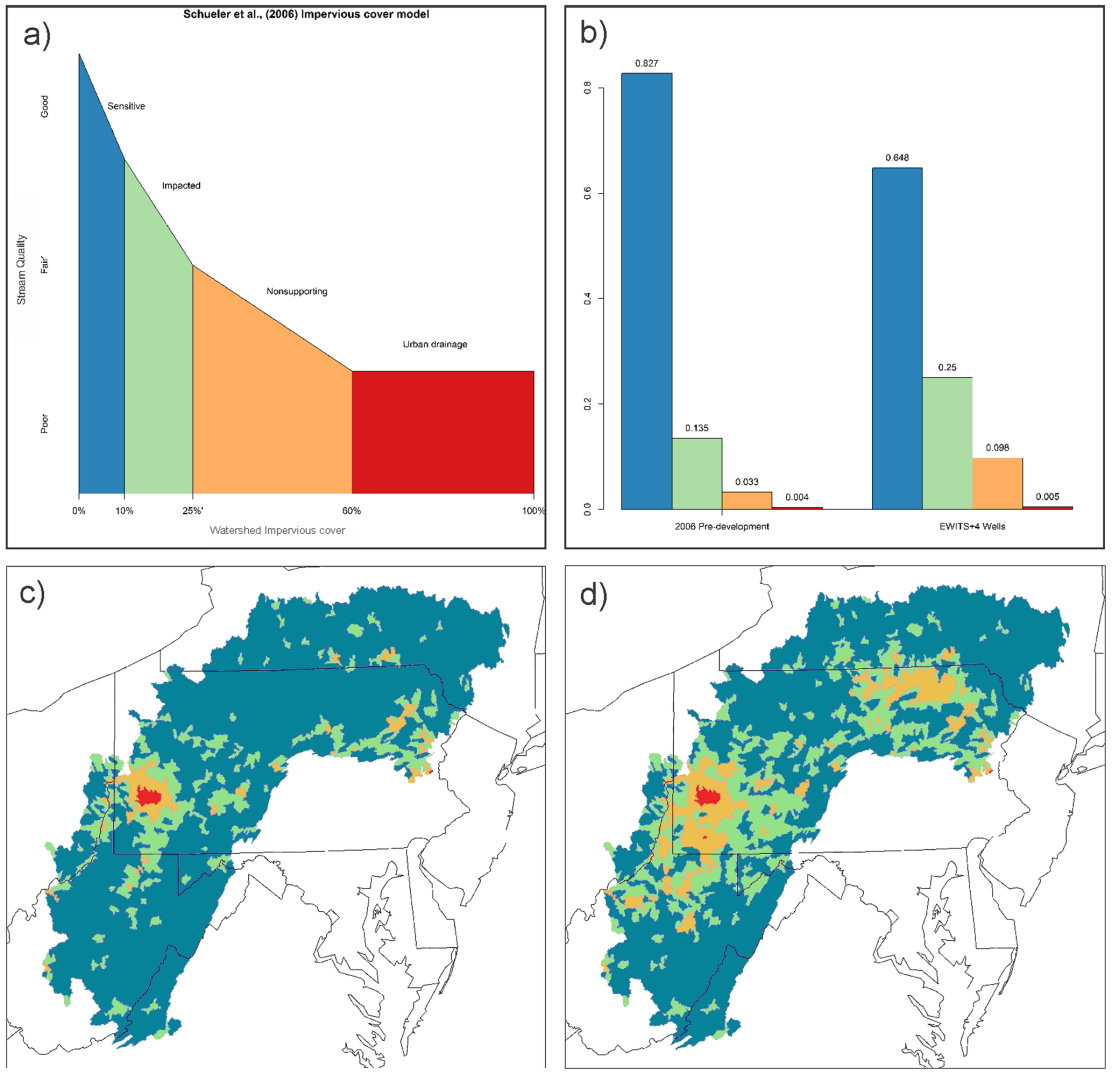
Impervious cover model at subwatershed-level; a) Graph of watershed impervious cover model (Schueler et al. 2009) representing classification of percent impervious surface. Colors in each impact class correspond to other panels in figure, b) bar graph showing percent subwatersheds in each class, c) 2006 “pre-development” subwatershed impacts, d) EWITS wind + 4 wells per pad subwatershed impacts.

After our projections of energy development, we can expect 26,501 new well pads and 10,798 new wind turbines. In addition to the 68,255 ha attributed to current shale gas and wind development, new gas pads and wind turbines will results in an additional 329,585 (EWITS+8 wells/pad scenario) – 603,278 (EWITS+4 wells/pad scenario) ha of impervious surface (Figure 3d), and drive 268,503–495,357 ha of deforestation. These impacts will result in changes that will affect conditions at the subwatershed level. For example, at present 0.4% (*n* = 9) of the subwatersheds are already classified as urban drainages and 3% (*n* = 67) as nonsupporting subwatersheds. Predicted energy development will result in up to a 171% (*n* = 130) increase of subwatersheds in the urban drainage and nonsupporting category (Figure 3). Under the highest cumulative impact scenario, 10% (*n* = 206) of the study area subwatersheds will be classified as either a nonsupporting or urban drainage (Figure 3). Our analysis also predicts that energy development will be disproportional in high-value watersheds (Figure 3). Three of the basin-level watersheds (Figure 1, watersheds: “02050”, “05020”, “05030”) will receive ∼85% of the increased surface disturbance forecasted by our modeling (Figure 3). We see that 36% of the predicted energy expansion footprint in our study area is expected to occur in watersheds that are in the top quartile in terms of intactness and water importance.

## Discussion

Our analysis indicates that in the Central Appalachians region, energy development will drive significant land use change, with shale gas development as the dominant driver of increased impervious surface and deforestation accounting for 94% of the projected footprint. This increase in surface disturbance and fragmentation will potentially impact the maintenance of biodiversity and the quality of surface water resources for ∼22 million people. The increase in energy production forecasted by our analysis may be compatible with biodiversity if properly sited, but will still pose a challenge for surface water resources, both because of the strong link between surface water quality and surface disturbance and because of the high value for water production for watersheds in the study area. Watersheds in the study area scored among the highest relative “water importance” in the country because of both the high level of water produced and the number of people that consume the water (all >60/100) [35]. Here we utilize the Impervious Cover Model (ICM) [51], [52], [59] to relay potential impacts as a result of projected energy sprawl. The ICM has been extensively tested with more than 250 different reports reinforcing the basic model (see for review [51], [52], [59]). However, the ICM is not intended to predict the precise score of individual surface water quality metrics and given the scale of our analysis, we do not attempt to make specific site-level predictions. We use the ICM to highlight areas where the cumulative impact of increased impervious cover and deforestation could result in decreased water quality, changes in hydrology and geomorphology, increased storm runoff, and sedimentation.

We focused our analysis on the impacts of surface disturbance on water quality from nonpoint sources, however, hydraulic fracturing (fracking) used to extract shale gas poses additional risk for water quality. Fracking involves the injection of a mix of water, chemicals and sand underground at high pressure to fracture rock and release the oil or gas [60]. Risks to surface water supplies include depletion of fresh water supplies, spills of fracking chemicals, leaks of flow back fluids that can include fracking chemicals once the well is completed and discharge of treated flow back fluids from wastewater facilities [13], [61]. For example, Olmsted et al. [13], showed that release of treated fracking wastewater increased chloride (CI) by 10–11% resulting in higher risk to surface water. Threats to underground water supplies can also result when wells are poorly constructed and when old oil and gas wells that have been capped serve as a migration corridor for fracking fluids used in new wells nearby [61]. While we did not assess additional risk associated specifically with fracking, our analysis can serve to highlight areas where additional attention should be placed to monitor potential fracking related impacts.

In contrast to traditional gas development, shale gas is developed with multiple horizontal wells that can reach out 1524 m or more from one well pad [60]. The footprint associated with shale gas development is typically bigger than conventional shallow gas plays [12]. The well pads are typically more expansive (averaging just over 1.21 ha compared to a small fraction of 0.40 ha), the water used to fracture wells is much greater (∼7–15 million liters vs. <500,000 liters), and the supporting infrastructure is much larger in scale (24″ diameter pipelines to gather gas from wells versus 2″ or 4″ pipelines in shallow fields). In addition, associated pipelines represent a significant source of fragmentation and, as our scenarios predict, can result in approximately half of the impervious surface associated with shale gas development. While the larger pad, greater water use, and more extensive infrastructure pose more challenges for conservation than shallow gas, the area “drained” by wells on each well pad is much larger than from shallow gas pads (200–400 ha versus 4–32 ha) since there are typically multiple lateral wells on a shale pad versus a single vertical well on a shallow gas pad [61]. The lateral reach of shale gas wells means there is more flexibility in where pads and infrastructure can be placed relative to shallow gas [12], [61]. This increased flexibility in placing shale gas infrastructure can be used to avoid or minimize impacts to natural habitats in comparison to more densely spaced shallow gas fields. Given this flexibility, our results can be used to facilitate ecologically appropriate siting of development, ensuring that key ecological features that could be impacted are highlighted so they can be preserved. Impacts to water quality can also be mitigated by attention to siting. Siting well pads and wind turbines to reduce potential runoff will therefore be critical, and will involve appropriate setbacks from streams and wetlands as well as avoiding development in steep slopes and grades. In addition, slowing the rate of development to allow targeted regulatory oversight for watersheds projected to experience increased development will be critical to allow balance between development objectives and conservation goals.

In marked contrast wind development may have less flexibility than shale gas development to adjust siting in ways that will mitigate potential impacts within our study area. Developing wind energy on disturbed lands rather than placing new developments within large and intact habitats would reduce cumulative impacts to wildlife [46]. Kiesecker et al. [46] found that there are over 14 times the amount of wind energy potential on disturbed lands needed to meet the DOE goal of generating 20% of the United States electricity with wind [8]. Despite the extensive wind resources across the U.S., the states comprising the majority of our study area (WV, PA, VA,) are unable to meet DOE projections within areas already disturbed [46]. In these states wind energy potential is largely restricted to ridge tops, which often make up the heart of the last remaining intact natural ecosystems [62]. In addition impacts associated with wind development will be influenced by the placement of transmission lines [47]. While our analysis takes into account the local disturbance associated with individual turbines we do not account for large scale transmission necessary to facilitate wind development. To fully understand and estimate impacts associated with wind development it will be critical to incorporate the effect of new transmission lines.

Our scenarios used the most current information to estimate shale gas and wind development patterns in this region [8], [27]. But the estimation of Marcellus Shale gas resources is highly uncertain, given the short production history. As additional data are released and the methodology for estimating the resource is refined, the ultimate estimated recovery for the Marcellus play may change. Also, as more wells are drilled over a broader area, and as operators optimize well spacing to account for evolving drilling practices, the assumption for average well spacing may also be revised. While several other studies have attempted to project future development scenarios within the Marcellus shale they have tended to focus on a subset of the area likely to be developed. Nonetheless these estimates are consistent with our estimate of the number of wells likely drilled. For example in their assessment of the economic impact of development in West Virginia the National Energy Technology Laboratory [25] project upwards of 40,000 new wells would be drilled by 2030. In a similar analysis focused on the economic impact of development in Pennsylvania, Considine et al. [24] project that upwards of 25,000 new wells would be drilled by 2020. The same can be said for our estimates of potential wind development. The estimation of wind development patterns is also highly uncertain, and will be influenced by a number of factors, i.e., investment in transmission, fuel costs and emission regulations [4], [45]. We utilized an aggressive scenario that emphasizes development of wind resources close to load centers, which likely represents an estimate on the high end of potential wind development for this region [49]. Given the current scope of our modeling efforts, accounting for local and global market demand at specific time-scale is unrealistic. Because of this, we are not assuming a specific time scale for development, but rather the full potential build-out of each resource. This gives us a general idea of potential impacts of undirected development independent of market fluctuations.

In a 2003 report to the World Bank, Dudley and Stolton [63] concluded that to control erosion and sediment, maintain water quality, and in some cases capture and store water, protecting forest in water catchment areas is “no longer a luxury but rather a necessity.” From an ecosystem services standpoint, the treatment cost of providing safe drinking water to urban areas increases dramatically with loss of forest [63]. In light of the new energy future in this landscape, as well as globally, understanding and mitigating the impacts of energy development will be one of the major challenges in the coming decade. Moving siting and mitigation decisions to a landscape scale rather than decisions made site by site, well by well will allow the regulators the ability to examine cumulative impacts like those discussed here [10].

## Conclusions

The Marcellus Shale represents one of the fastest growing shale deposits in the world. With both wind and shale gas projected to expand dramatically in coming decades predicting patterns and impacts in the Marcellus could serve as a model for development that is likely to be replicated globally. Already, Argentina, Australia, China, and Colombia have identified large shale gas deposits that are in the planning stages of development. The impacts from individual gas wells/wind turbines or even those of a single wind farm or gas field are likely to be manageable and compatible with broader landscape level conservation goals. Our analysis reveals it will be the cumulative impacts that pose the greatest challenge for landscape level conservation goals. Unfortunately assessment of environmental impacts are made well by well or gas field by gas field with little or no attempt to assess cumulative impacts [64]. Scenarios and scenario analysis have become popular approaches in pursuit of sustainable development [18]. However, they are little used, at least in any formal way, in environmental impact assessment (EIA). Fostering the use of scenario modeling, like the approach outlined here, can allow regulators to examine the potential consequences of development objectives quickly and inexpensively. We conclude by encouraging EIA practitioners to learn about the promise of scenario-based analysis and implement scenario-based methods so that EIA can become more effective in fostering sustainable development.
